# Nanotechnology and cancer: improving real-time monitoring and staging of bladder cancer with multimodal mesoporous silica nanoparticles

**DOI:** 10.1186/s12645-016-0015-8

**Published:** 2016-04-27

**Authors:** Sean K Sweeney, Yi Luo, Michael A O’Donnell, Jose Assouline

**Affiliations:** Department of Biomedical Engineering, University of Iowa, 1402 Seamans Center for the Engineering Arts and Sciences, Iowa City, IA 52242 USA; NanoMedTrix, LLC, 2500 Crosspark Road, Suite E119, Coralville, IA 52241-4710 USA; Department of Urology, University of Iowa, Roy J. and Lucille A. Carver College of Medicine, 3204 Medical Education Research Facility, 375 Newton Road, Iowa City, IA 52242 USA; Department of Urology, University of Iowa, Roy J. and Lucille A. Carver College of Medicine, 200 Hawkins Dr., Iowa City, IA 52242 USA

**Keywords:** Mesoporous silica nanoparticles, Transitional cell carcinoma, Bladder, Magnetic resonance imaging, Cystoscopy, Mouse orthotopic tumor model

## Abstract

**Background:**

Despite being one of the most common cancers, bladder cancer is largely inefficiently and inaccurately staged and monitored. Current imaging methods detect cancer only when it has reached “visible” size and has significantly disrupted the structure of the organ. By that time, thousands of cells will have proliferated and perhaps metastasized. Repeated biopsies and scans are necessary to determine the effect of therapy on cancer growth. In this report, we describe a novel approach based on multimodal nanoparticle contrast agent technology and its application to a preclinical animal model of bladder cancer. The innovation relies on the engineering core of mesoporous silica with specific scanning contrast properties and surface modification that include fluorescence and magnetic resonance imaging (MRI) contrast. The overall dimensions of the nano-device are preset at 80–180 nm, depending on composition with a pore size of 2 nm.

**Methods:**

To facilitate and expedite discoveries, we combined a well-known model of bladder cancer and our novel technology. We exposed nanoparticles to MB49 murine bladder cancer cells in vitro and found that 70 % of the cells were labeled by nanoparticles as measured by flow cytometry. The in vivo mouse model for bladder cancer is particularly well suited for T1- and T2-weighted MRI.

**Results:**

Under our experimental conditions, we demonstrate that the nanoparticles considerably improve tumor definition in terms of volumetric, intensity and structural characteristics. Important bladder tumor parameters can be ascertained, non-invasively, repetitively, and with great accuracy. Furthermore, since the particles are not biodegradable, repetitive injection is not required. This feature allows follow-up diagnostic evaluations during cancer treatment. Changes in MRI signals show that in situ uptake of free particles has predilection to tumor cells relative to normal bladder epithelium. The particle distribution within the tumors was corroborated by fluorescent microscopy of sections of excised bladders. In addition, MRI imaging revealed fibrous finger-like projections into the tumors where particles insinuated themselves deeply. This morphological characteristic was confirmed by fluorescence microscopy.

**Conclusions:**

These findings may present new options for therapeutic intervention. Ultimately, the combination of real-time and repeated MRI evaluation of the tumors enhanced by nanoparticle contrast may have the potential for translation into human clinical studies for tumor staging, therapeutic monitoring, and drug delivery.

## Background

Transitional Cell Carcinoma (TCC) of the bladder is the fifth most common malignancy in the United States (Siegel et al. 2015). Although the majority of human bladder cancer is superficial at the time of detection, the recurrence rate and the risk of progression to advanced disease are high (Hall et al. 2007; Kaufman et al. 2009; Faba et al. 2012; Nargund et al. 2012). Currently, the most common screening method is cystoscopy (Liu et al. 2012), which includes white light as well as fluorescent or narrow band cystoscopy (Kriegmair et al. 1994; Filbeck et al. 2002; Joudi and Konety 2004). The fluorescent and narrow band methods aim to highlight or differentiate the tumor from normal bladder epithelium wall using broad range/non-specific fluorescent dyes or stains (Filbeck et al. 2002; Joudi and Konety 2004). These generic labeling methods are not aimed at any particular molecule, but rather colorize the entire bladder with the hope that some differential labeling may occur. Macroscopic tumors can be seen with these methods, but smaller lesions may be missed (Zaak et al. 2005; Grossman et al. 2006). In addition, suspicious smaller lesions need biopsy for cancer staging, as cystoscopy alone remains insufficient (Cheng et al. 2000, 2009; Mitra et al. 2012).

The development of diagnostic tools for TCC has been slowed by the lack of an optimal animal model for the disease, due to the anatomical inaccessibility and the relative lack of molecular and bioinformatics data on the genetic diversity of the specific tumors. A variety of experimental models have been tested with mixed success, including mice (Chan et al. 2009; Zhang et al. 2015), rabbits (Nemoto et al. 1981), and primates (Cozzi et al. 2001). The current standard is a mouse orthotopic tumor model in which a known TCC cell line (MB49) is implanted following chemical disruption of the normal epithelium (Soloway et al. 1973; Bockholt et al. 2012; Newton et al. 2014). This cell line has similar features and replicates the pathobiology of human homologues (Luo et al. 2004). Although the presence of reporter genes has allowed for some measurements of non-invasive real-time tumor growth, no methods have been described capable of providing 3-dimensional details of growth, including heterogeneities within the tumor, in real time.

In addition to diagnostic tools, therapeutic options for TCC are limited by anatomical challenges. After initial resection, many patients undergo bacillus Calmette-Guerin (BCG) immunotherapy; however, the recurrence rate for BCG is up to 50 % (Hall et al. 2007; Faba et al. 2012). Furthermore, many patients cannot complete the therapy due to the risks of sepsis and other side effects (Lamm et al. 2000; Brausi et al. 2014). Thus, groups have attempted to package an attenuated form of the BCG cell wall skeleton into nanoparticles to more safely induce an immune response, with mixed results (Ochiai et al. 1983; Nakamura et al. 2014). Many other forms of nanoparticles are used as carriers of chemotherapeutics; (Connolly et al. 2012; Huang et al. 2012) these, however, often cannot be visualized/detected and have poor retention in the bladder. Very few examples exist of chemical agents that can be used both for detection and therapeutic solutions. For example, radioactive agents produce quality PET/SPECT images, (Hoilund-Carlsen et al. 2014; Bouchelouche and Choyke 2015) but have short half-lives, necessitating repeated doses for longitudinal studies, further increasing the patients’ exposure to ionizing radiation. Thus, there is a need for an advanced compound which would provide a good image using lowered-cost equipment (ultrasound, MRI), with no ionizing radiation and with good retention in the bladder for follow-up growth evaluation. The development of a single agent capable of providing sequentially valuable information from MRI as well as ultrasound contrast [image-guided surgical techniques (Nyland et al. 2002; Gkritsios et al. 2014)] would be highly desirable.

Mesoporous silica nanoparticles (MSNs) are often studied as vehicles for drug delivery due to their large ratios of pore volume and surface area to weight relative to other nanomaterials (Giri et al. 2005; Gruenhagen et al. 2005; Slowing et al. 2007; Chen et al. 2010; Benezra et al. 2011). Once injected, MSNs are bioinert, non-toxic and well-tolerated at clinically relevant concentrations (Slowing et al. 2006; Lu et al. 2008, 2010), unlike biodegradable polymers that can generate toxic byproducts (Athanasiou et al. 1996; Semete et al. 2010), or nanomaterials with reactive elements such as quantum dots (Kirchner et al. 2005; Liu et al. 2014). In addition, the use of MSNs in imaging applications is an area of active research. In particular, MSN are used to label and track cells through functionalization with fluorophores (Giri et al. 2005; Chen et al. 2010; Benezra et al. 2011), paramagnetic materials for magnetic resonance imaging (MRI) (Chen et al. 2010; Hsiao et al. 2008; Larsen et al. 2009), or electron dense materials for computed tomography (CT) (Chen et al. 2010; Luo et al. 2011).

Here, we describe the application of a novel MSN tool for noninvasive and longitudinal tracking of bladder cancer, using an established in vivo mouse model for the disease. Murine bladder cancer cells (MB49) were found to take up the MSN in vitro; in the mouse bladder, the MSN were taken up preferentially by tumor cells more readily than healthy bladder epithelium. The MSN were further functionalized with a peptide found to bind specifically to bladder cancer cells, thus improving specificity. The MSN facilitated the use of tumor imaging/staging in vivo using MRI, and ex vivo using fluorescent microscopy. This study presents the potential of these MSN to improve tumor visualization and staging, thereby improving patient outcomes.

## Methods

### Reagents

Gadolinium (III) chloride hexahydrate (GdCl_3_·6H_2_O), cetyltrimethylammonium bromide (CTAB, CH_3_(CH_2_)_15_N(CH_3_)_3_Br), diethylene glycol, tetraethoxysilane (TEOS), and (3-aminopropyl)trimethoxysilane (APTMS) were purchased from Alfa Aesar (Ward Hill, MA). Sodium hydroxide (NaOH) was purchased from VWR (Radnor, PA). Methanol, dimethyl sulfoxide (DMSO), and toluene were purchased from Fisher Scientific (Pittsburgh, PA). Tetramethylrhodamine isothiocyanate (TRITC) was purchased from Sigma-Aldrich (St. Louis, MO). 2-[Methoxy(polyethyleneoxy)propyl]trimethoxysilane was purchased from Gelest (Morrisville, PA). Dulbecco’s modified eagle medium (DMEM), Roswell Park Memorial Institute (RPMI) 1640 medium, fetal bovine serum (FBS), and penicillin–streptomycin (pen-strep) were purchased from Life Technologies (Grand Island, NY).

### Particle synthesis/characterization

First, a gadolinium oxide colloid was obtained following the previously reported synthesis (Bridot et al. 2007): GdCl_3_·6H_2_O (11.53 g) was dissolved in 200 mL of diethylene glycol at 60 °C overnight under vigorous stirring. Aqueous NaOH (7.5 mL, 3 M) was added and the solution was heated at 140 °C for 1 h and then at 180 °C for 4 h. The obtained transparent colloid of gadolinium oxide nanoparticles was stored at room temperature. CTAB (1.0 g, 2.745 mmol) was dissolved in nanopure water (480 g, 26.67 mol), followed by the addition of NaOH solution (2.0 M, 3.5 mL, 7.0 mmol). The mixture was heated to 80 °C for 1 h. To this clear solution, TEOS (4.7 g, 22.56 mmol) was added drop wise, followed by immediate addition of 1 mL of the gadolinium oxide colloid. The reaction was stirred vigorously at 80 °C for 2 h, then the CTAB surfactant was removed by Soxhlet extraction with methanol for 24 h and then dried under vacuum to obtain gadolinium oxide functionalized mesoporous silica nanoparticles (Gd_2_O_3_–MSN). Next, TRITC (5.7 mg, 0.0128 mmol) was reacted with APTMS (2.2345 μL, 0.0128 mmol) in DMSO for 2 h, and TRITC–Gd_2_O–MSN was prepared by grafting 0.05 mL of the resulting product on the previously synthesized Gd_2_O_3_–MSN (100 mg) in toluene under reflux for 24 h. The resulting solution was filtered and the obtained pink solid was washed with copious amount of methanol and then dried under vacuum. Finally, the particles were further functionalized with poly(ethylene glycol) (PEG) by grafting 2-[Methoxy(polyethyleneoxy)propyl] trimethoxysilane (0.2 mmol) on TRITC–Gd_2_O_3_–MSN (100 mg) in toluene under reflux for 24 h. The resulting solution was filtered and the obtained pink solid (PEG–TRITC–Gd_2_O_3_–MSN) was washed with copious amount of methanol and then dried under vacuum.

The materials were characterized by X-ray diffraction, using a Rigaku Ultima IV diffractometer, nitrogen sorption analysis in a Micromeritics ASAP 2020 surface area and porosity analyzer using the Brunauer-Emmett-Teller equation to calculate surface area and pore volume and the Barrett-Joyner-Halenda equation to calculate the pore size distribution. Dynamic light scattering (DLS) was used to obtain particle size distribution and zeta potential data, using the Malvern Zetasizer Nano ZS instrument. The materials were also visualized by transmission electron microscopy (TEM) by supporting samples on copper grids in a Tecnai G2 F20 microscope operating at 200 kV.

### Cell culture/labeling

Murine TCC cell line MB49 (Summerhayes and Franks 1979) and human TCC cell line T24 (Bubenik et al. 1973) are well-established; here, MB49 cells transfected with either green fluorescent protein (GFP) or luciferase (luc) reporter genes, and normal T24 cells were cultured in RPMI 1640 medium supplemented with 10 % FBS and pen-strep. Cells near confluence on tissue culture plastic were exposed to PEG–TRITC–Gd_2_O_3_–MSN at a concentration of 100 μg/mL for 16 h. Flow cytometry was used to measure the proportion of labeled MB49-GFP^+^ cells, while fluorescent microscopy was used to evaluate labeling on T24 cells. Prior to in vivo experiments, labeled and non-labeled cells were counted and the viability measured using trypan blue exclusion dye.

### In vivo tumor implantation

All procedures were performed according to NIH guidelines and previously approved by the Institutional Animal Care and Use Committee (IACUC). Intravesicular instillation of tumor cells was performed according to previously described methods (Luo et al. 2004). Female C57Bl/6 mice were anesthetized with a ketamine/xylazine mixture. The bladder was chemically burned by instillation of 5 μL 0.2 M silver nitrate, followed by rinsing with 100 μL phosphate buffered saline (PBS). A 50 μL suspension containing 5 × 10^5^ MB49-Luc^+^ cells in 50 % normal mouse serum was instilled into the bladder and retained for 1 h by catheter exclusion. Additional boluses of 1 × 10^5^ tumor cells were injected subcutaneously in the left and right flank. In initial experiments designed to determine maximum signal, cells were labeled with MSN prior to instillation; in later studies designed to be more clinically relevant, the tumor was established prior to instillation of free MSN particles (1 mg MSN/50 μL PBS). Negative control mice were instilled with sham (saline) injections, followed by instillation of free MSN following the same timing as tumor mice.

### Imaging/image processing

Images were acquired 24 h after instillation of particles to allow for the elimination of free particles through urination. *From 1*–*8* *days following tumor implantation,* in vivo fluorescence and luminescence were measured using the IVIS 200 with appropriate settings for detection of GFP, TRITC, and luciferase activity following intraperitoneal injection of 1.5 mg luciferin. MRI scans were acquired using the Unity/INOVA 4.7 T small animal scanner (Varian, Palo Alto, CA) with a 25-mm gradient RF coil and fast spin echo multislice (fsems) sequences with T1- or T2-weighting (T1-weighted scans: *T*_R_ = 800 ms, *T*_E_ = 15 ms, echo train length = 8, number of averages = 10; T2-weighted scans: *T*_R_ = 2000 ms, *T*_E_ = 15 ms, echo train length = 8, number of averages = 10). Typical image dimensions are 512 × 512 with 30 slices and a voxel size of 0.068 × 0.068 × 0.4 mm. Scans were saved as DICOM image stacks, which were converted to NIFTI format and preprocessed using a MATLAB routine developed in the lab, following a method of background removal Salvado et al. (2006a, b) and interpolation by reverse diffusion Salvado et al. (2006a, b). Using the free medical image processing software MIPAV, scans were normalized to one another by assigning a value of 1000 to the average intensity of fat adjacent to the kidneys, with all other values linearly interpolated between 0 and 1000. Volumes of interest (VOIs) were either manually drawn or semi-automatically selected using the “levelset VOI” tool in MIPAV.

### Histopathology and statistical analyses

Following the MRI scans, mice were euthanized by CO_2_ inhalation. The bladders were excised, embedded in OCT medium, flash frozen in liquid nitrogen and sectioned. A portion of the sections were mounted on glass slides without stain for use in fluorescence microscopy, while adjacent sections were stained with hematoxylin and eosin for histological analysis. Fluorescence images were acquired using standardized camera settings (Olympus DP70 camera, 1360 × 1024 resolution, and 15 s exposure time), while corresponding bright field images were acquired using the same resolution and the camera’s auto-exposure setting with color correction. The average red fluorescence intensity of each 10× field was quantified by isolating the red channel and using VOI tools to isolate regions of tissue from the background, using the bright field images as a reference for identifying the tissue and background. The average fluorescence intensity of each image was normalized by dividing it by the average intensity of the background. Comparisons of measurements between groups of mice were made using Student’s unpaired *T* test with an alpha level of 0.05 considered significant. Comparisons of different tumors within the same mouse were made using a two one-sided *t*-test (TOST) (Hong et al. 2015) with a significance level of *α* = 0.05 and a test margin of 0.05 (a unitless value of normalized MRI intensity), representing the average smallest difference between two intensities that our image processing technicians could discern with the naked eye.

## Results and discussion

### Particle characterization

**Fig. 1:**
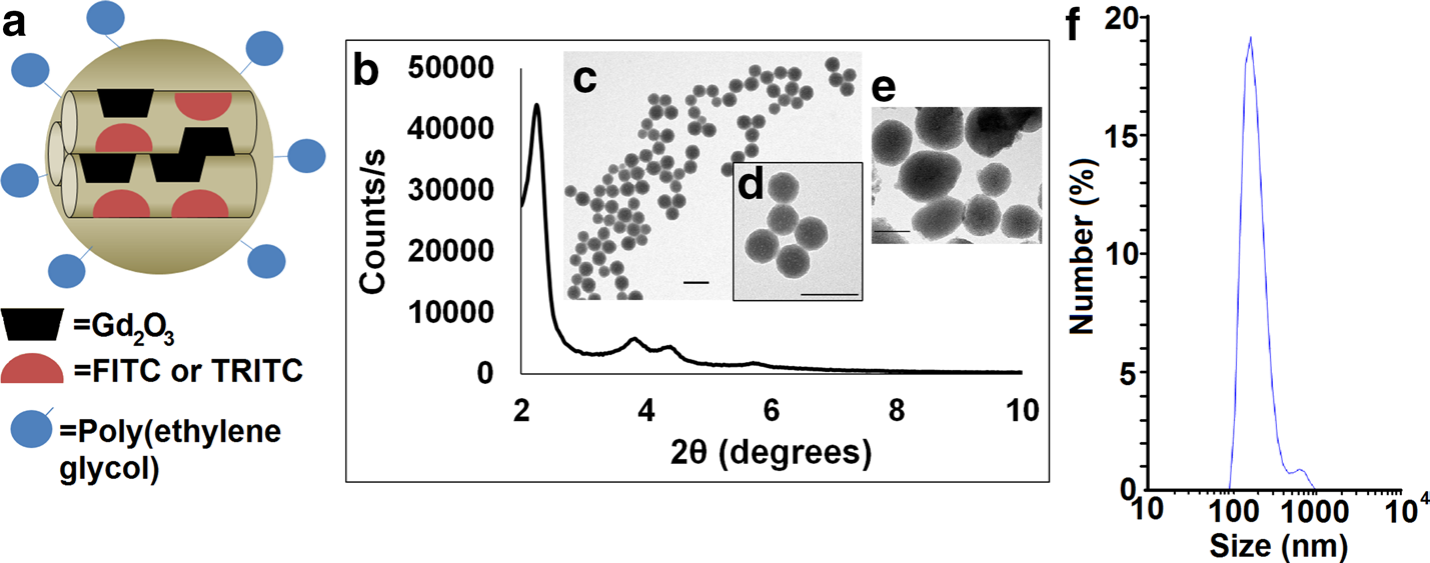
Characterization of PEG–TRITC–Gd_2_O_3_–MSN. **a** Schematic showing multifunctional MSN particle with Gd_2_O_3_ incorporated into silica matrix, and FITC or TRITC grafted onto the silica surface, followed by poly(ethylene glycol). **b** Powder X-ray diffraction patterns of TRITC–Gd_2_O_3_–MSN after surfactant removal. The intense peak at 2.5 2θ is characteristic of hexagonally arranged pores in MSN. **c** Transmission electron micrograph of synthesized MSN, with higher magnification *inset* (**d**), and fully synthesized PEG–TRITC–Gd_2_O_3_–MSN (**e**). Visualized particles had a uniform size distribution and showed no formation of aggregates. **f** Hydrodynamic size distribution of PEG-TRITC–Gd_2_O_3_–MSN as measured by dynamic light scattering. The primary peak has a median particle size of 187.3 nm. *Scale bars* indicate 100 nm

We synthesized multifunctional MSN for in vivo imaging of bladder tumors according to the schematic (Fig. 1a). By incorporating Gd_2_O_3_ into the silica matrix and covalently grafting FITC or TRITC onto the particle surface, the MSN is functionalized for fluorescent and MRI imaging modalities. Systematic characterization was performed at each step in the process of synthesizing the PEG–TRITC–Gd_2_O_3_–MSN. Powder XRD analysis confirmed hexagonally arranged mesopores in the diffraction pattern of the Gd_2_O_3_–MSN as evident by the intense d_100_, and well resolved d_110_ and d_200_ peaks characteristic for MSN (Fig. 1b). Transmission electron micrographs of the Gd_2_O_3_–MSN particles showed this pattern as well as uniform size distributions and good dispersibility with little aggregation (Fig. 1c–e, inset). Nitrogen sorption analysis of the TRITC–Gd_2_O_3_–MSN exhibited a Type-IV isotherm, typical of mesoporous materials, with a BET surface area of 710 m^2^g^−1^. The average pore diameter for TRITC–Gd_2_O_3_–MSN by BJH calculation is 24 Å. The fully synthesized PEG–TRITC–Gd_2_O_3_–MSN was characterized by DLS; the median hydrodynamic diameter of the particles was 187.3 nm, with 96.3 % of the particles falling within the primary distribution peak, spanning between 90 and 400 nm, resulting in a polydispersity index (PDI) of 0.535 (Fig. 1f). The number of particles greater than 400 nm (likely aggregated MSN) is less than 4 % of the total and does not have adverse experimental implications on the tagging of bladder tumors. Prior to use in tissue culture or in vivo, the fluorescence (FITC or TRITC) was measured using serial dilutions in a fluorimetric plate reader. In order to eliminate unbound fluorophores from the suspension, the particle suspensions were repeatedly rinsed in phosphate buffered saline and centrifuged until no detectable fluorescence was observed in the supernatant.

### Labeling/flow cytometry

**Fig. 2:**
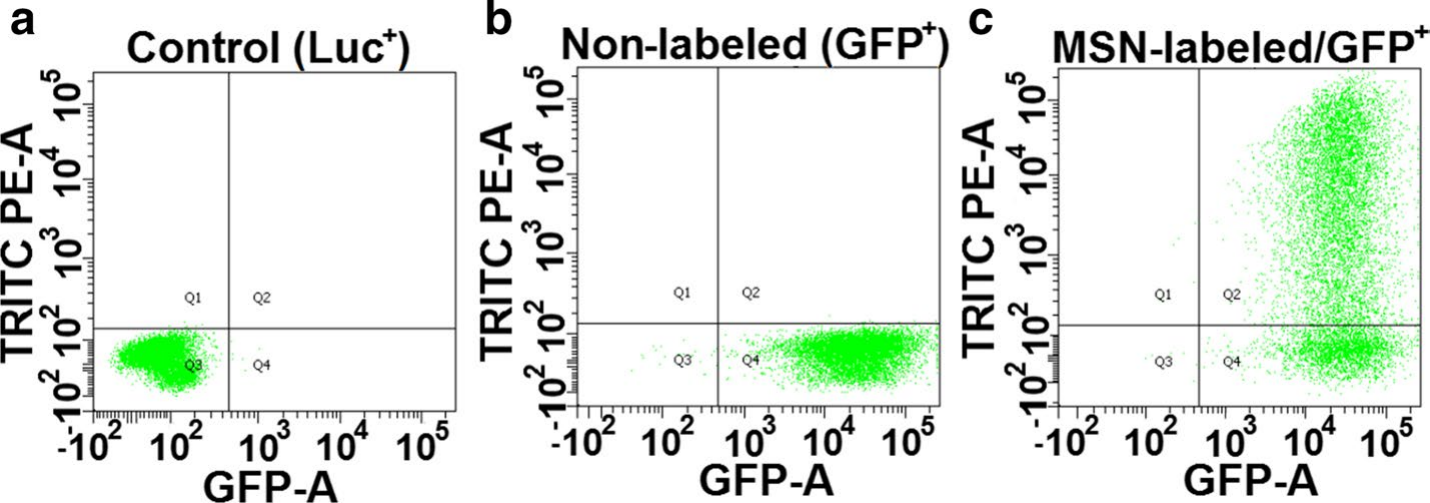
Functionalized mesoporous silica nanoparticles (MSN) are taken up by mouse bladder cancer cells. Flow cytometry for MB49 bladder cancer cells exposed to 100 μg/mL PEG–Gd_2_O_3_–TRITC–MSN. **a** MB49-Luc^+^ cells were used as negative controls for both GFP and TRITC. **b** MB49-GFP^+^ cells prior to labeling were 99.8 % GFP^+^/TRITC^−^. **c** After labeling, 69.6 % of the cells were found to be GFP^+^/TRITC^+^, with a range of levels of rhodamine fluorescence, indicating some cells picked up more MSN particles than others

Particle uptake by TCC cells was determined by flow cytometry. GFP^−^/TRITC^−^ cells were used to establish the threshold for detection (Fig. 2a) while the GFP^+^/TRITC^−^ cells were used to confirm the efficiency of GFP transfection, which was found to be 99.8 % (Fig. 2b). In the final sample, 69.6 % of the cells were found to be GFP^+^/TRITC^+^ (Fig. 2c), with a wide range of levels of rhodamine fluorescence, indicating some cells picked up more MSN particles than others.

### In vivo fluorescence

**Fig. 3:**
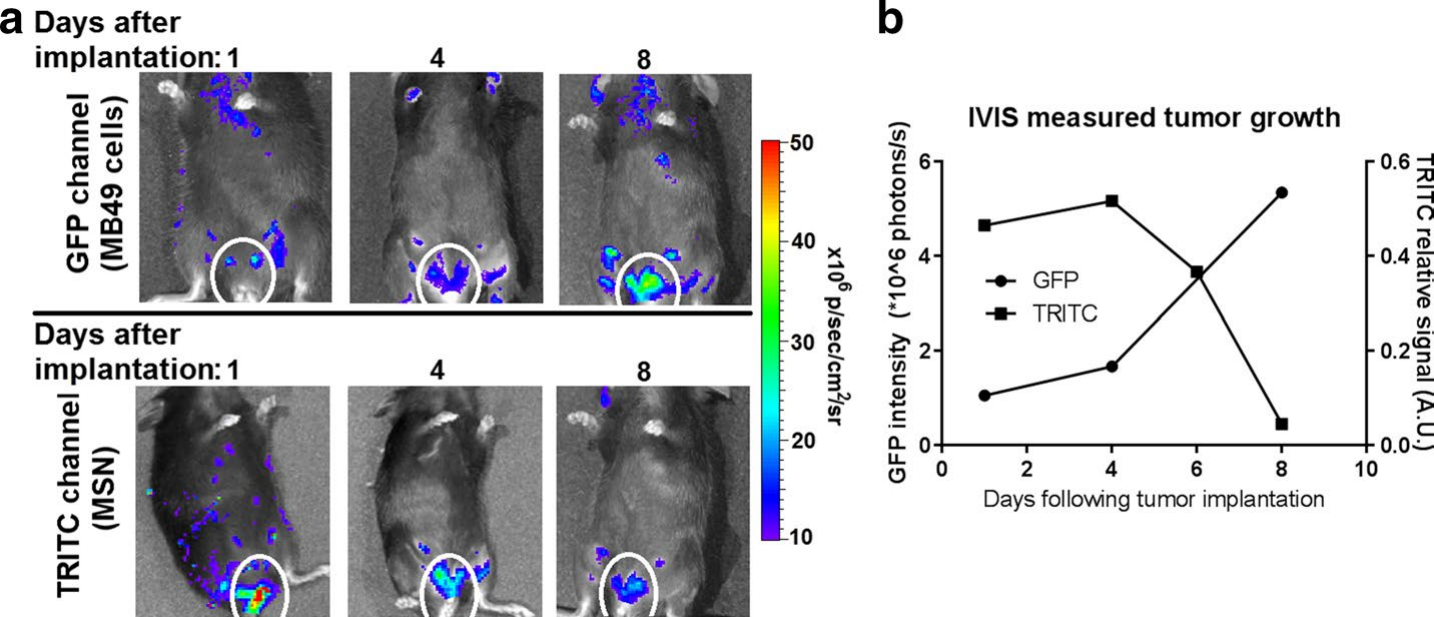
In vivo measurement of growth in tumors labeled with PEG–CF_3_–Gd_2_O_3_–TRITC–MSN. **a** IVIS imaging of GFP and TRITC fluorescence in a mouse injected with labeled MB49-GFP^+^ cells. The top row indicates GFP signal arising from the cells. The day 1 signal is low because the number of implanted cells is small; when the tumor reaches a significant size, a large signal is detected. The second row shows the signal from PEG–CF3–Gd2O3–TRITC–MSN. The signal is detectable immediately, despite a small number of tumor cells implanted. MB49 cells engulf the particles where they remain in the cytoplasm. Following each mitotic event, the cytoplasm is split between the daughter cells, thus dividing the signal in half with each cellular division. In **b**, we confirm a relative and progressive attenuation of the TRITC signal. Taken together, the increase in GFP fluorescence as the tumor grows coincides with the diffusion of TRITC fluorescence. Thus, the labeling provides an additional level of early detection not found in unlabeled MB49 tumors

Implantation of PEG–TRITC–Gd_2_O_3_–MSN-labeled MB49-Luc^+^ and MB49-GFP^+^ cells was confirmed using in vivo luminescence and fluorescence (Fig. 3). Using the standard detection methods (luciferin luminescence or GFP fluorescence), the tumor was detectable within 6–8 days in vivo, consistent with previous results. Using the MSN for detection (TRITC fluorescence), the tumors were detectable in 1–4 days (Fig. 3b). The growth rate was consistent with that of unlabeled tumor cells, indicating that the particles are not inhibitive of tumor growth.

### MRI imaging

We implemented two tumor labeling approaches, representing incremental stages moving towards translational significance. In the first, we labeled cells prior to implantation to determine the maximal MRI signal attainable. Subsequently, we implanted the tumor cells first, labeling them in vivo, to represent a clinically relevant scenario.

1. To determine the maximum MRI signal, MB49-Luc^+^ cells were labeled with PEG–Gd_2_O_3_–TRITC–MSN prior to instillation in the bladder to obtain the best possible contrast in MRI. Tumors were also implanted in the left and right flank, using labeled and non-labeled cells, respectively, as controls. With T1-weighting, a hyperintensity was observed for tumors comprised labeled cells relative to non-labeled cells (Fig. 4a). After segmentation, the normalized MRI values of labeled (161.2 ± 11.8) versus non-labeled (143.3 ± 12.9) flank tumors were compared using the TOST test for equivalency with a test margin of 0.05 and found to be non-equivalent at a significant (*p* < 0.0001) level (Fig. 4b). Further, the tumor instilled into the bladder (159.5 ± 12.8), also comprised MSN-labeled cells, was found to be equivalent to the flank tumor comprised labeled cells (*p* > 0.05) and non-equivalent to the tumor comprised non-labeled cells (*p* < 0.001). This initial test also confirmed that the process of labeling MB49 cells with MSN did not alter their ability to engraft, either subcutaneously or in the bladder. Three-dimensional rendering of the tumor shows infiltration of the bladder wall, potentially metastasizing to nearby tissues (Fig. 4c). This represents clinically important data for tumor staging.

**Fig. 4:**
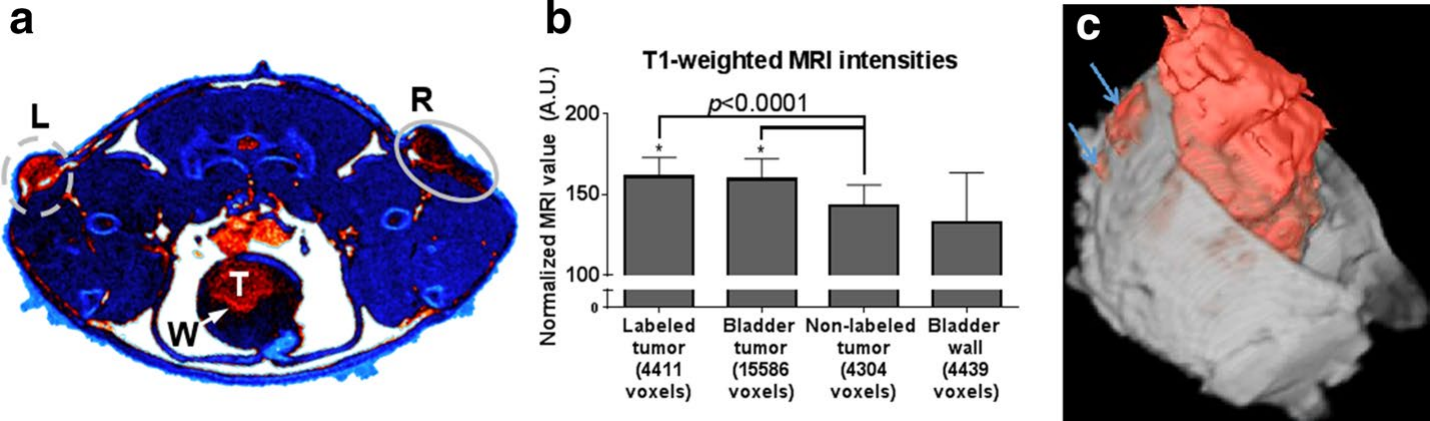
Optimal/maximum binding of MSN: bladder cancer cells were labeled (70 %) prior to injection into mouse and then MRI performed. **a** A pseudocolor slice of T1-weighted axial MRI data showing definite intensity difference 8 days post-injection between the two subcutaneous injections in the thigh (particle labeled cells in the left flank, L, and non-labeled cells in the right flank, R) as well as the tumor within the bladder (in situ) (T) and bladder wall (W). **b** The measured MRI values for each region shown. *Error bars* indicate standard deviation; * *asterisk* when compared using the two one-sided *T*-test of equivalence, the intensity of labeled tumor in the left flank and bladder were not equivalent to the non-labeled tumor and bladder wall (*p* < 0.0001). **c** A 3-D virtual cystoscopy rendering of the same tumor in (**a**) with the tumor identified by fuzzy C-means segmentation (*red*). Unique features: *arrows* indicate regions where the tumor has invaded the bladder wall, an important pathological milestone
2. To represent a more clinically relevant scenario, we subsequently injected MB49 tumors to establish the tumor, then after 8 days we labeled in vivo via intravesicular instillation of colloidal PEG–Gd_2_O_3_–TRITC–MSN particles (1 mg MSN in 50 μL PBS). MRI scans showed immediately before and 1 day after MSN addition to determine the qualitative effects of the MSN on tumor characterization as well as the quantitative changes in T1- and T2-weighted MRI signal for bladder tumor relative to normal bladder epithelium. A representative 2-D slice of T2-weighted MRI of the same tumor is shown before (Fig. 5a) and 24 h after (Fig. 5d) addition of MSN particles. Each pair of images is shown after normalization, using equivalent input–output color maps. Additional 3-D renderings of the tumor in situ (Fig. 5b, e) and segmented (Fig. 5c, f) reveal unique features within the tumor, including finger-like projections which have a different consistency than the bulk of the tumor and can be traced along the outer boundary. Quantitatively, the animals with tumors showed a much larger magnitude of change in normalized MRI value after administration of particles than the bladder walls of non-tumor, sham-injected negative control mice (Fig. 6). This result provides evidence that in vivo tumors take up MSN particles with a higher affinity than normal bladder epithelium. The projections were found to be considerably hypointense in these renderings of T2-weighted scans, indicative that in larger tumors, a subset of cells have an especially high affinity for the MSN.

**Fig. 5:**
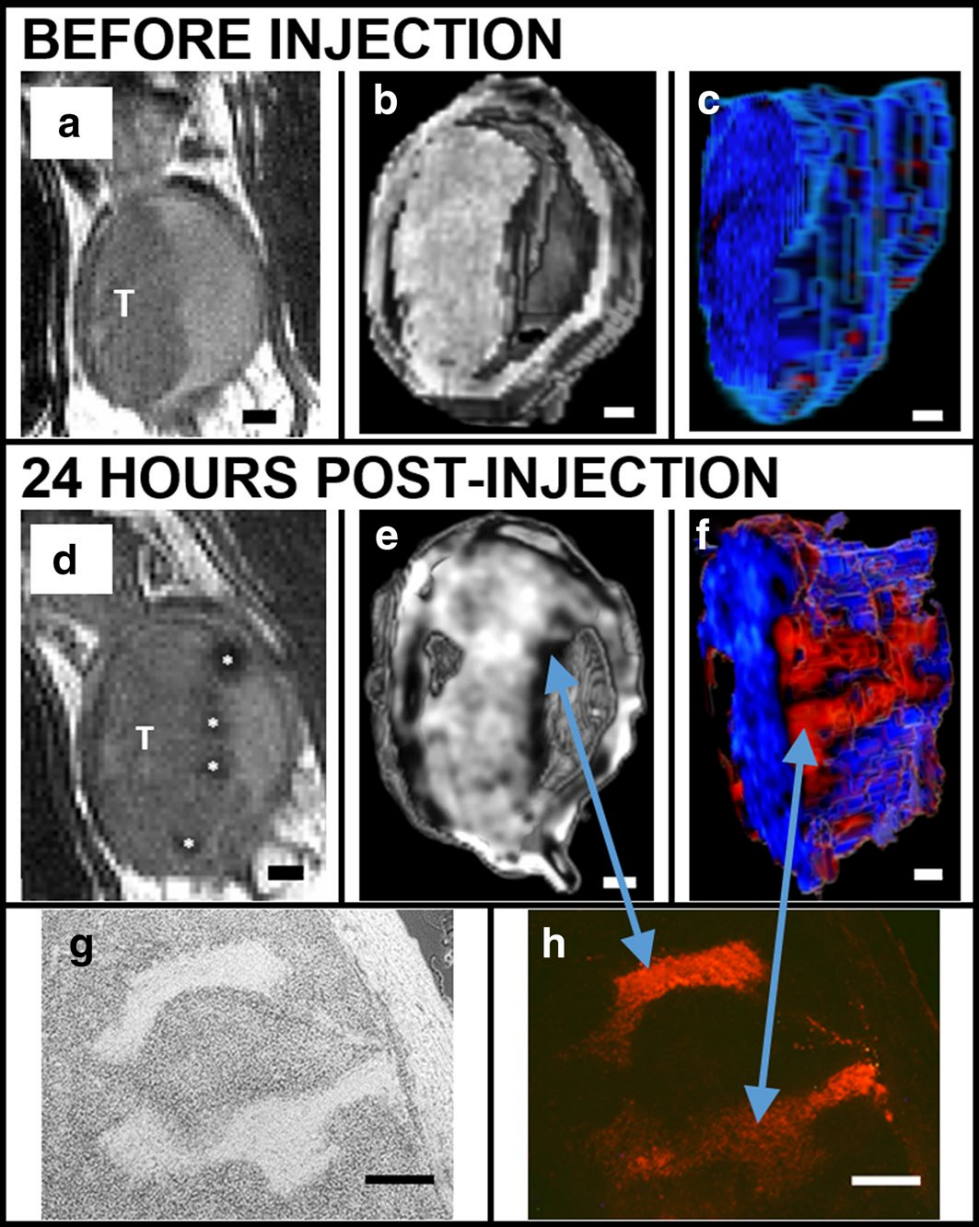
Evaluation of increased contrast and histopatholgical benefits of our nanoparticle technology. MSN bind preferentially to bladder cancer cells relative to normal bladder epithelia in vivo as shown in a series of renderings of T2-weighted MRI scans acquired before (**a**–**c**) and after (**d**–**f**) intravesicular instillation of Gd_2_O_3_–TRITC–MSN. **a** and **d** 2-dimensional grayscale view, the tumor (T) is shown before and after injection of particles; note clear labeling of the tumor surface * *asterisk*. (**b**, **e**) 3-dimensional rendering provides further evidence of the particle distribution on the tumor surface. **c**, **f** Here the tumor is segmented and rendered with a pseudocolor map. Finger-like projections are revealed which are not observed before the injection of particles (**c**). Histology confirms anatomical observations and particle penetrations in the structures within the tumor: *bright field* (**g**) and fluorescent microscopy (**h**). *Scale bars* 1 mm (**a**–**f**); 250 μm (**g**, **h**)

**Fig. 6:**
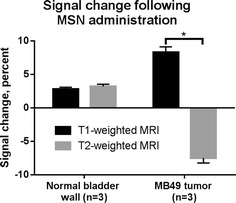
T_1_ and T_2_ signal changes specific to the in vivo bladder tumor as the result of particle injections. The normalized average MRI intensities of bladder tumor and normal (sham-injected, negative control) bladder wall were measured before and after administration of MSN. The result was an increase in apparent T_1_ intensity and a decrease in T_2_ intensity in tumor bladders, compared with minimal change in the normal bladder wall (*−*p* < 0.0001)

### Histological analyses

**Fig. 7:**
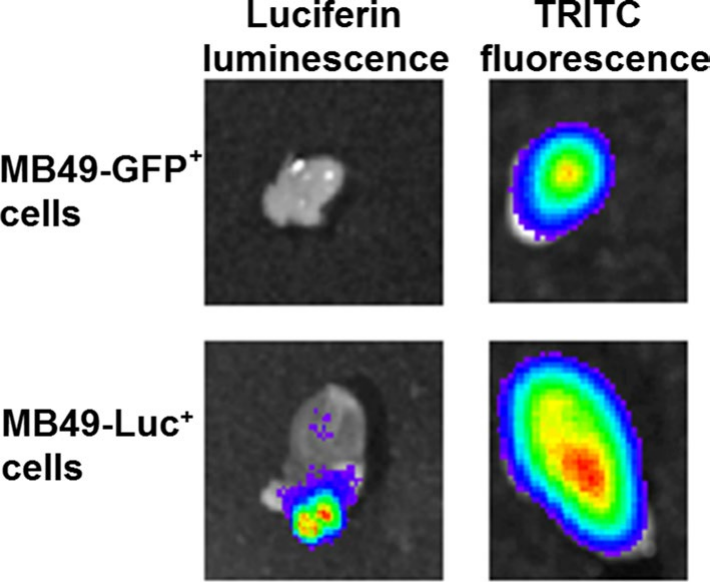
IVIS images of ex vivo bladders under luciferase luminescence and DsRed fluorescence. Ten days after implantation of labeled MB49 cells, one mouse from each group was sacrificed and the bladders were excised along with the kidneys and portions of lung for comparison. Luminescence was only observed in the bladder of the mouse injected with MB49-Luc^+^ cells, while both the MB49-Luc^+^ and MB49-GFP^+^ tumors were positive for TRITC fluorescence, indicative of the retention of PEG–CF_3_–Gd_2_O_3_–TRITC–MSN

**Fig. 8:**
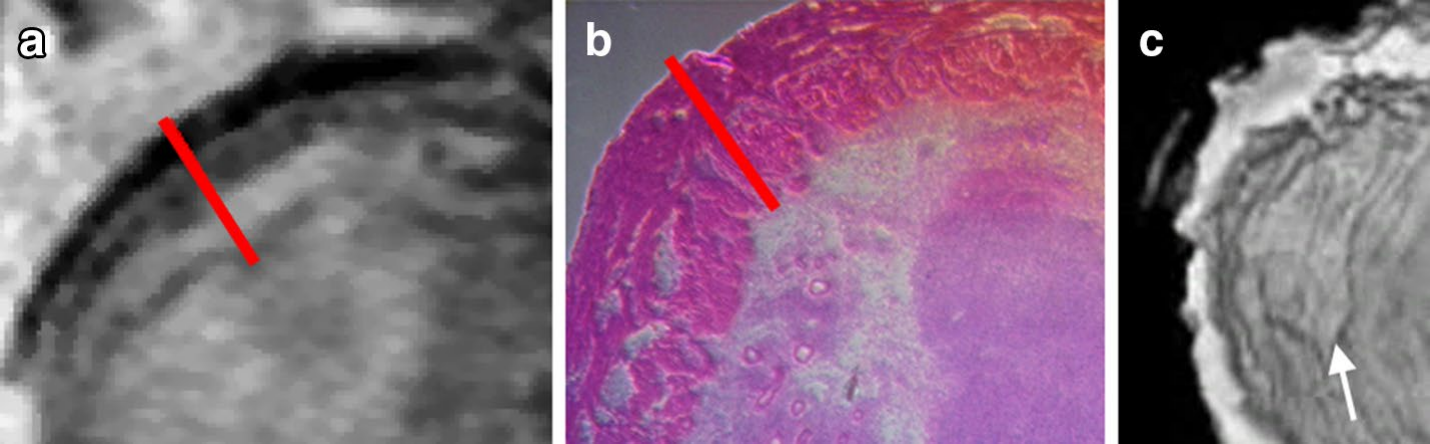
Multimodal evaluation of bladder wall in a tumor model. **a** T2-weighted image of bladder wall. Bladder wall thickness (*red line*) was calculated to be ~1.00 mm or approximately 17 pixels. **b** Histological section of same bladder after excision. Bladder wall thickness was also measured to be ~1.00 mm. **c** The many folds of the bladder epithelium can also be visualized in 3-dimensions, as seen in this rendering (*arrow*)

To validate and confirm MRI observations, we process tissues for histological evaluation. The bladders were excised after the final MRI scans, re-scanned for whole tissue fluorescence signal using the IVIS system, and then serially sectioned. Gross anatomical observations using IVIS show, as expected, only the Luc^+^ tumor showing luciferase luminescence, while both the GFP^+^ and Luc^+^ tumor have positive fluorescence in the red channel, indicating retention of the PEG–Gd_2_O_3_–TRITC–MSN (Fig. 7). The finger-like projections seen in MRI (Fig. 5f) were observed to be regions of higher cell density/faster growth (Fig. 5g), and are consistent with observations made of TCC in humans (Cheng et al. 2009). Additionally, the fluorescence micrograph (Fig. 5h) corroborates the MRI scan, showing that these are the areas of greatest particle uptake. In both the T1- and T2-weighted MRI scans, we observed clear improvements in boundary delineation. This allowed for the in vivo evaluation of features such as the bladder wall with a level of detail approaching that of histological sections (Fig. 8). Based on measurements collected from both MRI and histological sections, we calculated the thickness of the bladder wall to be approximately 1.00 mm, corresponding to 17 pixels at our achievable resolution.

This report documents the findings on an orthogonal mouse model, and the potential for translation to clinical use of a novel nanoparticle-based technology. This approach may improve diagnostic and potentially therapeutic outcomes in TCC of the bladder. We are unaware of clinically relevant gold standards to use for a comparative basis. Clinical approaches include ImmunoCyt/uCyt™, a fluorescent test that uses three monoclonal antibodies (Vergara-Lluri et al. 2014), and UroVysion™, which is an in situ hybridization test using four different probes to different chromosomes (Seideman et al. 2015). We are aware of the benefits of Hexvix, or hexyl aminolevulinate, which is similar to a chemical found naturally in the body and contains porphyrins (Martoccia et al. 2014). The underlying principle of this approach is that cancer cells absorb this substance faster than healthy cells and turn fluorescent pink when the cystoscope light changes from white to blue. To date, we have not seen a report of cystoscopy-based approaches used in murine models.

Using the well-established MB49 murine line of bladder cancer cells, the uptake of our particles was measured in vitro, followed by non-invasive detection of labeled, implanted tumors in vivo using magnetic resonance imaging. In a pre-clinical animal model for bladder cancer, free particles were instilled in bladders with pre-established tumors. We demonstrate that the localization of the particles highlighted to the tumor helped delineate its edges and other features which were not otherwise detectable. We identified finger-like projections-areas which are consistent with the literature and are believed to be connective cellularized tissue (Cheng et al. 2000). These tumor penetrating structures displayed a higher particle uptake than the surrounding tumor. It is tempting to envision the potential therapeutic applications of the particles as they are able to access the inner cellular components of the tumor.

Two major hindrances to the recovery and poor prognostics of this form of cancer is the delay in detection of the cancer and its subsequent propensity to rapidly metastasize to adjacent tissue (Faba et al. 2012; Kaufman 2006). Although TCC is among the most deadly forms of cancer, the methods for diagnosis, including fluorescent and colored dyes that “paint” the bladder wall and delineate the tumor for use with cystoscopic techniques, however, are lacking in both depth of penetration and accuracy (Liu et al. 2012; Bryan et al. 2014). Because the depth of tumor penetration is difficult to grade through cystoscopic means alone, the bladder is often removed as a precautionary measure, to the severe detriment of quality of life for the patient (Kaufman et al. 2009; Dalbagni et al. 2009; Gakis et al. 2013). Our results show a novel option based on a core of MSN for multimodal use in cancer diagnostics. In addition, the potential is clear for the material to be used therapeutically, as a carrier of anti-tumor agents. The engineered nanoparticles are made to improve visualization of the tumor through T1- and T2-weighted computational MRI techniques.

Using the well-established MB49 murine TCC line in culture, we found 69.6 % of cells to be labeled with our PEG–Gd_2_O_3_–TRITC–MSN, with most cells fluorescing between 2 and 3 orders of magnitude above the detection threshold for the flow cytometry instrument. The particles were well tolerated by the cells; viability of labeled cells remained high (over 90 %) as indicated by trypan blue exclusion dye measurement and by virtue of the successful engraftment of labeled tumor cells in vivo. The manner of uptake has not yet been characterized, though from the literature and our own observation it is most likely that particles are engulfed by phagocytosis. The uptake mechanism of similarly sized and functionalized MSN has been characterized by other groups as co-localized with endosomes (Slowing et al. 2006; Hsiao et al. 2008). These studies have examined the involvement of clathrin-coated pits, using inhibition of clathrin to show reduced uptake (Huang et al. 2005), and lysosomes, using fluorescent lysosomal tracking dyes (Slowing et al. 2006). Additional determination of uptake awaits future experimentation with specific cell signaling molecules on the MSN surface.

The current standard pre-clinical model for TCC is the mouse orthotopic model in which MB49 murine TCC cells are implanted onto a chemically disrupted bladder epithelium (Luo et al. 2004). Although the model presents a number of advantages, i.e., it reliably creates tumors which closely mirror the histological picture of human TCC, we found that the tumor growth varies greatly depending on the quality of the chemical disruption and the number of cells that bind to it. In addition, the small size of this animal model does not lend itself to cystoscopy or other clinically relevant screening techniques. In this report, we show that by combining the animal model with the image improvement offered by our MSN, we can use small animals (mice) and create valuable virtual cystoscopy. Our early experiments involved labeling of cells with our particles prior to implantation in order to optimize our imaging parameters, and to show that labeled cells will engraft and grow in a similar manner to non-labeled tumor cells. Control injections of labeled and non-labeled cells implanted subcutaneously in either thigh of the mouse showed similar growth characteristics, indicating that cell labeling alone did not adversely affect tumor growth or implantation. Importantly, our technology allows for the non-invasive detection of histological features. Within the bladder, the tumor boundary was more easily delineated, and was observed to cross the bladder wall. These crucial data regarding depth of tumor penetration are impossible to obtain clinically through cystoscopy alone ( Cheng et al. 2000, 2009; Mitra et al. 2012).

Our experimentation strategy was first to establish a bladder tumor model in which nanoparticles would be most visible and then refine our approach to develop a method with more clinical relevance where the tumor was initiated in the bladder, then followed by injection and binding in vivo of free MSN 8 days later. Interestingly, prior to the instillation of particles, the MRI scan shows a very homogeneous tumor which reflects the uniformity of the MB49 cells in culture. Only after administration of particles are unique heterogeneities revealed, which are correlated histologically. This includes the formation of finger-like projections, forming largely around the boundaries of the bulk tumor. These regions appear to be more fibroblastic in morphology, with a denser matrix of connective tissue than the bulk tumor, as evidenced by the higher relative amount of eosin staining. Furthermore, these regions have a higher affinity for our MSN than the bulk tumor; this is consistent with our observations, and those of other groups, (Hsiao et al. 2008; Selvan et al. 2010) that MSN are readily engulfed by fibroblast cells. To the best of our knowledge, nothing is published regarding the heterogeneity of MB49 tumors in situ, though groups have reported the presence of finger-like projections in human clinical pathological specimens. (Cheng et al. 2000).

The typical clinical tool for bladder cancer diagnosis is white light or fluorescent cystoscopy, used in conjunction with a form of dye that improves definition of the tumor boundaries (Kriegmair et al. 1994; Filbeck et al. 2002; Joudi and Konety 2004). Clinically, MRI virtual cystoscopy of the bladder is less commonly used, (Raza and Jhaveri 2012) and although gadolinium-based contrast agents are sometimes administered intravenously prior to scanning, specific agents that bind tumors are not yet available. Our results indicate that intravesicular instillation of MSN particles improves differentiation of the tumor in MRI; the signal obtained from normal bladder wall is unaffected by particle instillation, whereas the signal of the tumor increases under T1-weighted MRI, and decreases under T2-weighted MRI (Fig. 6). This is consistent with the accumulation of gadolinium-based contrast agents in tumors, and corroborated by the difference observed in fluorescence signals from excised bladders of tumor and normal mice (Fig. 5). In normal bladder epithelia, the particles that were retained appeared to be discretized in macrophages, whereas the tumor tissue possessed a brighter, more widespread fluorescence throughout, or in the case of more heterogeneous tumors, spread among a particular subpopulation of the tumor.

In this study, taking into account the consideration that clinical efficacy of using nanoparticles may be hindered or diminished due to bladder epithelium becoming hyperpermeable in TCC bladders (Brown et al. 1993; Gontero et al. 2004). The immediate implications being that particles may bind to non-tumor regions in a cancerous bladder, thus leading to overestimation of the true tumor size. We showed that has not been the case in our study, as we clearly demonstrate that our particles have a predilection for cancerous tissue. Further improvement will have to wait for the development of specific targeting strategies.

With respect to image quality improvement, our current small animal scanner has a magnetic field of 4.7 T and is capable of in-plane image resolutions below 100 μm. At this resolution, we detected patterns in MRI corresponding to layers observed in histological sections, and visualized folding patterns within the bladder 3-dimensionally (Fig. 8). With the advent of MRI systems with magnets as large as 7 T and more powerful gradient coils, both in small animal and clinical use, we anticipate an improvement in signal/contrast for the same MSN formulation.

Thus, there is a need to improve with higher-specificity molecules. In addition to advances in imaging technology, the obtained signal can be further improved by increasing the specificity of the particles for bladder cancer cells. The particles presented thus far have not been functionalized for cell specificity; the specificity we observe is a result of exploiting the morphological differences between normal and tumor cells in the bladder. Whereas healthy/intact epithelium is defined by tight gap junctions and cell adhesion molecules only at the basement membrane, diseased (cancerous/inflamed) epithelium divides rapidly, has looser gap junctions, and overexpresses adhesion molecules, such as EpCAM (Bryan et al. 2014; Kowalski et al. 2010) integrins, (Knowles et al. 2013) laminin, cadherins, (Gontero et al. 2004) and other surface molecules (Parker et al. 2005; Tagaya et al. 2014). Due to these features, diseased epithelium has a higher affinity for foreign objects than normal epithelia. To improve upon this, our colleagues have identified a short peptide sequence using a phage display library with a high affinity for a wide range of bladder cancer cells across multiple species including mouse, dog, and human, and with little affinity for normal bladder epithelia. We intend to enhance the specificity of our particles through their functionalization with this peptide. To that end, we have developed a new particle capable of binding proteins/peptides through coupling chemistry (Hermanson 2008).

An additional desirable property of MSN particles which was not directly addressed in this study would be to have uptake and delivery of soluble materials within their porous structure. Many have successfully demonstrated delivery of bioactive agents in vitro using MSN (Giri et al. 2005; Gruenhagen et al. 2005; Slowing et al. 2007; Chen et al. 2010). The increased complexity of delivery in vivo makes it a more challenging scenario (Chan and Lin 2015; Wang et al. 2015). However, if some of these challenges can be overcome in the harsh chemical environment of the bladder, cancer treatment can be vastly improved. Whereas most chemotherapeutics are quickly eliminated from the bladder via urination (Grabnar et al. 2006; GuhaSarkar and Banerjee 2010), particles loaded with chemotherapeutics and retained by tumor cells will remain in the bladder for a longer time. Similarly, BCG immunotherapy can be improved through longer retention times, thus improving the depth of drug delivery, rather than simply removing the outermost cells a few layers at a time as current therapies tend to do (Kaufman 2006; Kim and Steinberg 2001).

## Conclusions

This study represents a unique example of the synergistic use of MRI, nanoparticles and computational imaging to visualize TCC in a mouse model in 3 dimensions, as a prototype for similar use in human TCC. Our approach allows for a significantly improved detailed window into the pathogenesis of the tumor, including invasion of the bladder wall and heterogeneities within the tumor. These pathological hallmarks are frequently observed in human-excised tissue. Currently, MRI 3-D reconstruction of the bladder is less common than traditional cystoscopic evaluation despite the fact that it is less invasive than cystoscopy, and important observations, including tumor staging, may be missed. The challenge in translating this technology to humans, where the tumor will be much smaller relative to the entire bladder at the time of imaging, will be improving the specificity for TCC to obtain the strongest signal possible. However, we predict that through additional research, a combination of MRI and fluorescent cystoscopy aided by MSN will make it possible to more accurately assess human TCC, and ultimately, if caught at a sufficiently early stage, improve patient prognosis.
